# Versorgungsaspekte der Elektrokonvulsionstherapie: Analyse der externen Zuweisungen an ein universitäres Zentrum

**DOI:** 10.1007/s00115-022-01360-9

**Published:** 2022-08-11

**Authors:** Isabel Methfessel, Michael Belz, Fabienne Bühler, David Zilles-Wegner

**Affiliations:** Klinik für Psychiatrie und Psychotherapie, Universitätsmedizin Göttingen, Von-Siebold-Str. 5, 37075 Göttingen, Deutschland

**Keywords:** EKT, Versorgungsforschung, Zuweisungsprozess, Regional, Leitlinien, ECT, Healthcare research, Allocation process, Regional, Guidelines

## Abstract

**Hintergrund:**

Die Anwendungshäufigkeit der Elektrokonvulsionstherapie (EKT) in Deutschland variiert stark in Abhängigkeit von der regionalen Verfügbarkeit. Teilweise wird dieses Versorgungsdefizit durch Zuweisungen an EKT-durchführende Kliniken kompensiert, was jedoch durch dort verfügbare Ressourcen limitiert ist.

**Ziel der Arbeit:**

Untersuchung der externen Zuweisungen zur EKT am Beispiel der Universitätsmedizin Göttingen. Analysiert werden sollen die Zuweiserstruktur, die Patientencharakteristika, die leitliniengerechte Pharmakotherapie vor Indikationsstellung zur EKT sowie das Therapieoutcome im Falle einer Behandlung mit EKT.

**Material und Methoden:**

Externe Anmeldungen zur EKT wurden über ein Jahr systematisch erfasst und retrospektiv ausgewertet. Neben der deskriptiven Darstellung der Daten erfolgte der Abgleich pharmakologischer Vorbehandlungen mit den aktuellen Leitlinienempfehlungen. Das Therapieoutcome nach durchgeführter EKT wurde mittels des klinischen Gesamteindrucks (CGI-I) bestimmt.

**Ergebnisse:**

Für *N* = 52 Patienten erfolgte die Anfrage zur Übernahme, davon kamen 82,7 % aus dem stationären Setting und aus einer Entfernung von bis zu 300 km. Unipolare Depressionen (57,7 %) und Störungen aus dem Schizophreniespektrum (36,5 %) waren die häufigsten Diagnosen. Vor Zuweisung erfolgte in der Mehrheit der Fälle mindestens eine leitliniengerechte Vorbehandlung. Bei 18 Patienten wurde eine EKT in unserem Haus durchgeführt, von diesen zeigten 72,7 % ein gutes bis sehr gutes Ansprechen.

**Diskussion:**

Anzahl und Radius der Zuweisungen zeigen einen hohen ungedeckten Bedarf in der Versorgung mit EKT und damit einen eingeschränkten Zugang zu einer evidenzbasierten und leitlinienempfohlenen Therapie. Im Sinne einer heimatnahen Behandlung ist anzustreben, EKT als Therapieangebot an mehr Kliniken zu etablieren. Auch bei externen Zuweisungen und damit verbundenen, zum Teil erheblichen Verzögerungen ist die Ansprechrate diagnoseübergreifend gut.

## Abstract (en)

**Background:**

The application of ECT in Germany varies widely depending on regional availability. This shortfall in ECT supply is partly compensated via referrals to hospitals with ECT services, yet restricted by limited resources in these clinics.

**Objective:**

External referrals for ECT were investigated at the University Medical Center Göttingen. We analyzed the referring institutions, patient characteristics, pharmacotherapy according to current guidelines before indications for ECT, and clinical outcome in cases of treatment with ECT.

**Material and methods:**

All external referrals were systematically recorded and retrospectively evaluated for the time span of 1 year. Besides descriptive presentation of the data, pharmacological pretreatment was compared with the current guideline recommendations. We used overall clinical impression (CGI-I) to determine the treatment response post-ECT.

**Results:**

External referrals were made for *N* = 52 patients, 82.7% of whom were from the inpatient setting and from a distance of up to 300 km. The most common diagnoses were unipolar depression (57.7%), followed by schizophrenia spectrum (36.5%). Prior to referral, at least one guideline-based pretreatment was given in the majority of cases. ECT was performed in 18 patients in our hospital, of whom 72.7% showed a good to very good response.

**Conclusion:**

Both numbers and radius of external referrals indicate a high unmet need for ECT and thus limited access to this evidence-based and guideline-recommended therapy. As treatment close to home should be the goal, more hospitals are needed to establish (or expand) ECT services; however, even with considerable delays which are often associated with external referrals, the response rate is good across all diagnoses.

## Hintergrund und Fragestellung

Die Elektrokonvulsionstherapie (EKT) ist ein hochwirksames und sicheres Therapieverfahren depressiver und psychotischer Erkrankungen [1–3].

Hierbei wird unter Kurznarkose und der Gabe von Muskelrelaxation mittels Strom ein generalisierter zerebraler Anfall ausgelöst [1]. Die Anwendungshäufigkeit der EKT variiert stark im internationalen sowie europäischen Vergleich [2]: Während z. B. in Schweden im Jahr 2013 41 Patienten pro 100.000 Einwohner mit EKT behandelt wurden [3], waren es in Deutschland im Jahr 2016 lediglich 6,9 pro 100.000 Einwohner [4].

Dennoch hat sich die Anwendung der EKT in Deutschland zwischen 2008 und 2016 auf rund 5700 Patienten pro Jahr etwa verdoppelt. Zugleich stieg die Zahl der Patienten, die explizit zur Durchführung der EKT an Kliniken mit entsprechendem Angebot verlegt wurden, überproportional an [4]. Darin spiegelt sich wider, dass die absolute Anzahl der Kliniken, die EKT durchführen (ca. 45 %), im selben Zeitraum konstant geblieben ist. Die Anzahl der in den jeweiligen Kliniken durchgeführten EKT-Behandlungen variiert hierbei stark.

Durch aktuelle Empfehlungen in den Leitlinien für unipolare Depression [5], bipolare Störungen [6] und Schizophrenie [7] ist zu erwarten, dass sich der Trend zur vermehrten Anwendung der EKT in Zukunft fortsetzt, was durch noch unveröffentlichte Analysen von Routinedaten untermauert wird [8].

Überregionale Zuweisungen sind bei bestehender Indikation zur EKT und fehlendem eigenem Angebot einerseits zu begrüßen, widersprechen andererseits jedoch dem Prinzip der regionalen und wohnortnahen Versorgung [9] und bringen praktische Schwierigkeiten mit sich. So kann es für die zuweisenden Kliniken schwierig sein, eine aufnehmende EKT-Klinik zu finden und Patienten erfolgreich zu vermitteln. Vor der Aufnahme eines Patienten kann es, vor allem wenn dieser nicht aus dem Einzugsgebiet stammt, zu langen Wartezeiten kommen. Zudem ist in vielen Fällen im Anschluss an die akute Behandlung eine längerfristige Erhaltungs-EKT indiziert [10, 11]. Dafür erhalten die Patienten in verschiedenen Zeitintervallen – in der Regel zwischen einer und sechs Wochen [12, 13] – einzelne EKT-Behandlungen. Nach Entlassung der Patienten aus der stationären Akutbehandlung kann eine fehlende regionale Versorgung mit EKT aufgrund weiter Transportwege sowie limitierter Ressourcen an den EKT-durchführenden Kliniken zu Versorgungslücken führen. Wird deshalb auf die Erhaltungstherapie verzichtet, sind die Patienten innerhalb der ersten sechs bzw. zwölf Monate (je nach Studie) einem hohen Rezidivrisiko (zwischen 44 und 49 %) ausgesetzt [14–16].

Die Leitlinien für unipolare Depression [5], bipolare Störungen [6] und Schizophrenie [7] empfehlen jeweils die EKT bei Therapieresistenz (siehe Tab. 1). Eine entsprechende Pharmakotherapieresistenz stellt mit die häufigste Indikation zur Durchführung einer EKT im klinischen Alltag dar. Zur Indikationsstellung ist daher eine Überprüfung der vorangegangenen (Pharmako‑)Therapie wichtig, zumal die EKT als personal- und zeitintensives Verfahren auch in den durchführenden Kliniken eine limitierte Ressource darstellt.

**Table 1:** 

Anwendungsempfehlungen für die EKT	Empfehlungsgrad
*Unipolare Depression* „EKT soll bei schweren, vital bedrohlichen oder therapieresistenten depressiven Episoden als Behandlungsalternative in Betracht gezogen werden“	A
*Schizophrenie* „Bei eindeutiger medikamentöser Behandlungsresistenz nach adäquater Therapie in ausreichender Dosis und Zeitdauer, sollte eine EKT zur Augmentierung […] angeboten werden“	B
*Bipolare Depression* „Die Elektrokonvulsionstherapie (EKT) sollte zur Behandlung schwerer und therapieresistenter depressiver Episoden im Rahmen einer Bipolaren Störung eingesetzt werden“	B

Die Klinik für Psychiatrie und Psychotherapie der Universitätsmedizin Göttingen führt in der Regel bis zu 45 EKT-Behandlungen pro Woche durch. Externe Zuweisungen erfolgen dabei standardisiert über ein Formular, auf dem für die Indikationsstellung relevante Informationen wie Diagnosen und Vorbehandlungen der Patienten erfasst werden. Um die Versorgungssituation mit EKT näher zu untersuchen, führten wir eine Analyse der in einem 12-Monats-Zeitraum erfolgten externen Anmeldungen zur EKT durch.

Ziele waren dabei die Analyse (1) der Zuweiser, (2) der angemeldeten Patienten, (3) der leitliniengerechten Pharmakotherapie vor Indikationsstellung und (4) der Wirksamkeit der EKT in dieser spezifischen Population extern zugewiesener Patienten.

## Methodik

### Stichprobe und Studiendesign

Es wurden alle externen Anmeldungen zur EKT an unserer Klinik im Zeitraum vom 1. Juli 2020 bis einschließlich 30. Juni 2021 ausgewertet. Die Erfassung der Daten des Anmeldeformulars sowie ggf. weiterer Daten (Arztbriefe, Medikamentenanamnese) erfolgte als Bestandteil des Zuweisungsprozesses und zur Indikationsstellung routinemäßig. Insgesamt handelte es sich um die Datensätze von *N* = 52 Patienten. Für die Datenauswertung liegt ein positives Ethikvotum vor (Antrag Nr. 24/7/21).

### Datenerhebung

Über ein Anmeldeformular auf unserer Homepage [17] sowie etwaige weitere Quellen (Entlassungsbriefe, Medikamentenlisten) wurden neben Alter und Geschlecht detaillierte Angaben zum klinischen Verlauf vor Anmeldung, soweit verfügbar, erfasst. Hierzu zählten psychiatrische Diagnosen, Erkrankungsalter und Krankheitsdauer, somatische Vorerkrankungen, pharmakologische Vorbehandlungen inklusive Art sowie Dosis und Dauer der Medikation. Weiterhin wurden das derzeitige Behandlungssetting sowie die Rechtsgrundlage der aktuellen Behandlung erfragt. Das Therapieansprechen der letztlich an unserer Klinik behandelten Fälle wurde mittels einer Rating-Skala zum klinischen Gesamteindruck aus den Arztbriefen extrahiert.

### Datenerfassung und -auswertung

Die Datenerfassung und -auswertung erfolgte mittels IBM SPSS Statistics 28 (IBM Corporation, Armonk, NY, USA) [18]. Die deskriptive Darstellung der Zuweiser sowie Patienten erfolgte über Mittelwerte (M), Standardabweichungen (SD), Häufigkeiten und Prozentwerte.

Für die Analyse der leitliniengerechten Pharmakotherapie wurden konkrete Empfehlungen zur Pharmakotherapie aus den aktuell gültigen Leitlinien für die Diagnosen unipolare Depression, bipolare Störung sowie Schizophrenie [5–7] extrahiert und mit den Vorbehandlungen der Patienten abgeglichen.

Anhand der siebenstufigen „Clinical Global Impression – Improvement Scale“ (CGI‑I; [19]) wurde eine Veränderung des klinischen Gesamteindrucks durch die EKT-Behandlung ermittelt (von 1 = „sehr stark verbessert“ bis 7 = „sehr stark verschlechtert“).

## Ergebnisse

### Zuweiserdaten und Zuweisungsprozess

**Figure 1:**
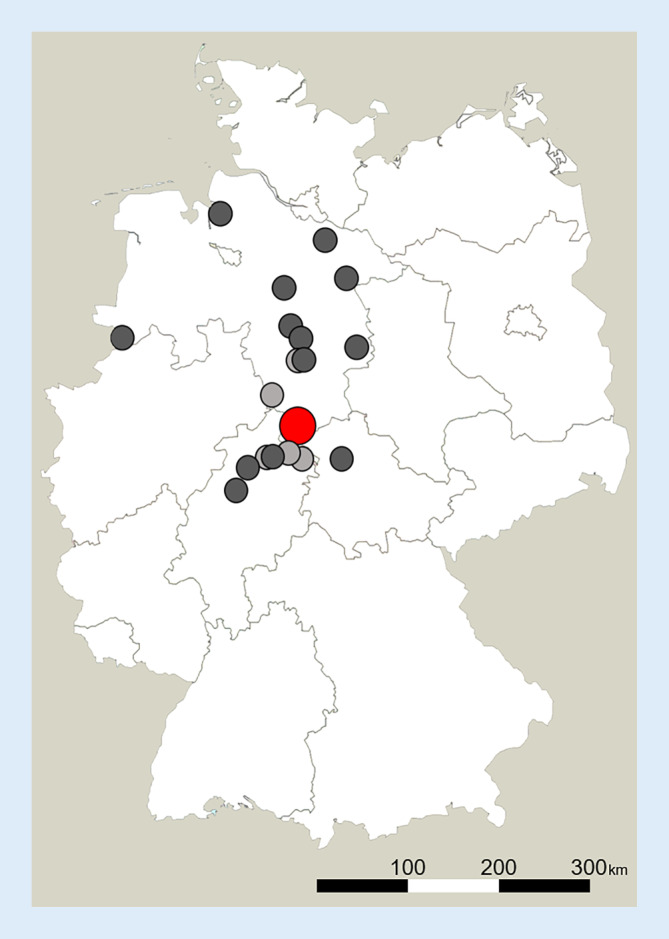

Innerhalb eines Jahres wurden *N* = 52 Patienten von extern zur EKT angemeldet. Die Zuweisungen erfolgten aus fünf Bundesländern: Niedersachsen (*n* = 34, 65,4 %), Hessen (*n* = 14, 26,9 %), Thüringen (*n* = 2, 3,8 %), Bremen und Hamburg (jeweils *n* = 1, 1,9 %). Die zuweisenden Kliniken/Praxen waren im Median 120 km Fahrstrecke von Göttingen entfernt (min. = 1 km; max. = 300 km, Interquartilsabstand [IQR; Verteilungsbreite der mittleren 50 % einer Stichprobe] = 68). 31 der 52 angemeldeten Patienten (59,1 %) wurden aus einer Distanz mehr als 100 km von der Universitätsklinik Göttingen entfernt zugewiesen. Von den 18 Patienten, die schließlich eine EKT erhielten, wurden 11 (61,1 %) aus einer Distanz mehr als 100 km von der Klinik entfernt aufgenommen. Für eine grafische Darstellung der Zuweisungen nach Bundesländern siehe Abb. 1. Bei der überwiegenden Mehrheit der Patienten (*n* = 43, 82,7 %) erfolgte die Zuweisung aus einem stationären Setting.

**Figure 2:**
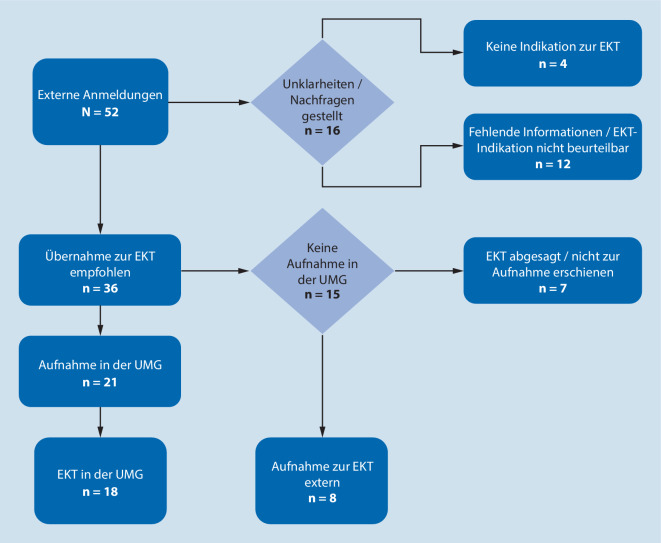

Für eine Übersicht des Zuweisungsprozesses siehe Abb. 2. Bei *n* = 36 der 52 (69,2 %) angemeldeten Patienten wurde eine Übernahme zur EKT empfohlen. Von diesen 36 Patienten wurden *n* = 21 (58,3 %) tatsächlich übernommen und bei *n* = 18 Patienten eine EKT in unserem Haus durchgeführt. Bei den weiteren 3 Patienten (teil-)remittierte die depressive Symptomatik vor Beginn der geplanten und dann obsoleten EKT. Von den 15 weiteren Patienten, denen eine Übernahme angeboten wurde, erfolgte bei *n* = 8 die Aufnahme zur EKT in einer Klinik mit kürzerer Distanz oder früherer Behandlungsmöglichkeit; *n* = 7 sagten vor erfolgter Übernahme ab oder erschienen nicht zur Aufnahme.

Bei *n* = 16 (30,8 %) der angemeldeten Patienten konnte anhand der initial zur Verfügung gestellten Unterlagen keine klare EKT-Indikation gestellt werden. In diesen Fällen erfolgten zum Teil Rückfragen und das Angebot eines direkten kollegialen Austauschs. Bei *n* = 4 Patienten bestand nach Prüfung der Unterlagen und Rücksprache mit den behandelnden Ärzten keine EKT-Indikation. In diesen Fällen wurden Behandlungsmöglichkeiten zur EKT im Rahmen einer persönlichen Rücksprache ausgetauscht.

### Patientencharakteristika

Von den *N* = 52 zugewiesenen Patienten waren *n* = 32 weiblich (61,5 %). Die Patienten waren im Mittel 53,0 Jahre alt (SD = 16,50). Die Mehrheit hatte die Diagnose einer unipolaren Depression (*n* = 30, 57,7 %), gefolgt von *n* = 19 (36,5 %) Patienten mit Störungen aus dem Schizophreniespektrum (*n* = 11 paranoide Schizophrenie, *n* = 5 katatone Schizophrenie, *n* = 3 schizoaffektive Störung). Drei Patienten (5,8 %) wurden mit einer biopolaren depressiven Störung zugewiesen.

### Pharmakologische Vorbehandlungen

Eine Pharmakotherapieresistenz stellte den häufigsten Grund für eine Zuweisung zur EKT dar. Der Abgleich der aufgeführten Vorbehandlungen zugewiesener Patienten mit Therapieempfehlungen aus den jeweiligen Leitlinien zeigte ein sehr heterogenes Bild.

Es stellte sich heraus, dass bei der Mehrheit der Patienten mindestens ein leitliniengerechter Behandlungsansatz bei Therapieresistenz verfolgt wurde. So wurde bei 80,0 % der unipolar depressiven Patienten ein Augmentationsversuch unternommen. Patienten mit Diagnosen aus dem Schizophreniespektrum erhielten in 94,7 % der Fälle eine Umstellung auf ein Antipsychotikum mit einem anderen Rezeptorprofil, in 73,7 % der Fälle einen Therapieversuch mit Clozapin. Bei 100 % der bipolar depressiven Patienten bestand im Vorfeld eine Phasenprophylaxe.

In den meisten Fällen war zu erkennen, dass zahlreiche medikamentöse Behandlungen versucht wurden. So erhielten alle unipolar depressiven Patienten im Vorfeld mindestens 2 unterschiedliche Antidepressiva (max. Anzahl 14; M = 5,43; SD = 2,89), 18 (von 19) Patienten mit psychotischen Erkrankungen mindestens 2 unterschiedliche Antipsychotika (max. Anzahl 13; M = 6,14; SD = 3,14). Die Qualität der durchgeführten Behandlungen war in vielen Fällen nur eingeschränkt beurteilbar, da Angaben zur Therapiedauer oder zum Medikamentenspiegel nicht verfügbar waren. Die ausführlichen Ergebnisse des Abgleichs der Vorbehandlungen mit den Leitlinienempfehlungen sind in Tab. 2 dargestellt.

**Table 2:** 

Therapieempfehlungen/empfohlenes Vorgehen bei Therapieresistenz	Empfehlungsgrad	Nachvollziehbar umgesetzt bei wie viel Prozent der Patienten vor Zuweisung (%)
*Unipolare Depression (n* *=* *30; *[5]*)*		
Therapiedauer mindestens 4 Wochen	KKP	43,3
Plasmaspiegelkontrolle	KKP	40,0
Augmentation	B	80,0
Augmentation mit Lithium	B	50,0
Augmentation mit Lithium in ausreichender Länge und Dosis	–	30,0
Antidepressivahochdosistherapie	0	20,0
Kombination von Mianserin oder Mirtazapin mit einem SSRI oder einem TZA	KKP	26,7
*Schizophrenie (n* *=* *19; *[7]*)*		
Therapiedauer mindestens 2 Wochen	A	42,1
Wechsel auf ein Antipsychotikum mit einem anderen Rezeptorbindungsprofil	KKP	94,7
Plasmaspiegelkontrolle	KKP	57,9
Behandlungsversuch mit Clozapin bei Behandlungsresistenz	A	73,7
Clozapinspiegel von mind. 350 ng/ml	B	26,3
*Bipolare Depression (n* *=* *3;* [6]*)*		
Etablierung einer Phasenprophylaxe	KKP	100,0
Optimierung der Phasenprophylaxe bezüglich Dosis und ggf. Serumspiegel	KKP	66,7
Therapiedauer mind. 3 Wochen	KKP	33,3
Quetiapin als Monotherapie zur Akutbehandlung	A	33,3

### Patienten mit EKT-Behandlung

**Figure 3:**
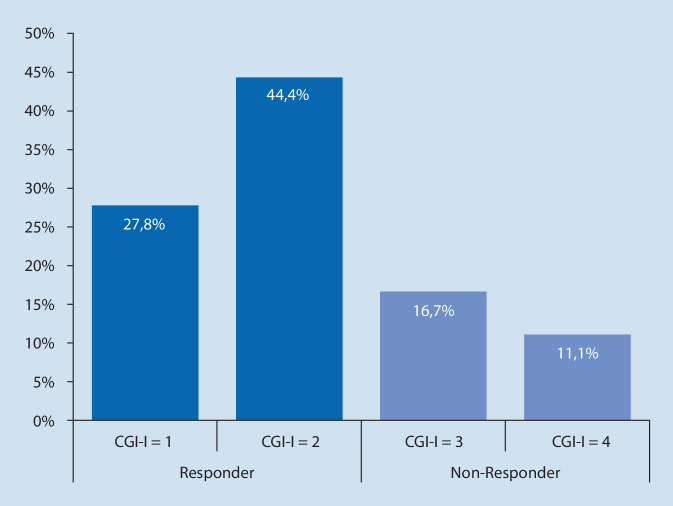

Von den *n* = 18 Patienten, die mit EKT behandelt wurden, waren *n* = 10 (55,5 %) unipolar depressiv, *n* = 6 (33,3 %) hatten eine Diagnose aus dem Schizophreniespektrum und *n* = 2 (11,1 %) waren bipolar depressiv. Insgesamt zeigten *n* = 13 (72,2 %) Patienten ein gutes Ansprechen (Abb. 3): Der klinische Gesamtzustand wurde als „sehr stark verbessert“ oder „stark verbessert“ beschrieben, was einer Bewertung von 1 oder 2 auf der CGI-I-Skala entspricht. Kein Patient verschlechterte sich. Fünf Patienten zeigten lediglich eine leichte oder keine Veränderung (Wert 3 oder 4), was als Non-Response definiert wurde. Siehe Tab. 3 für eine Darstellung der Response vs. Non-Response für die einzelnen Diagnosegruppen sowie Abb. 3 für die Gesamtgruppe. Die mittlere Wartezeit gerechnet ab Datum der Anmeldung bis zur ersten durchgeführten EKT-Behandlung betrug 56,6 Tage (SD 13,69).

**Table 3:** 

	Diagnose		
	Unipolare Depression (*n* = 10)	Schizophrenie/schizoaffektiv (*n* = 6)	Bipolare Depression (*n* = 2)
Response	*n* = 7 (70,0 %)	*n* = 4 (66,7 %)	*n* = 2 (100,0 %)
Non-Response	*n* = 3 (30,0 %)	*n* = 2 (33,3 %)	*n* = 0 (0,0 %)

Response definiert als Wert von ≤ 2 auf der CGI-I-Skala; Non-Response definiert als Wert ≥ 3 auf der CGI-I-Skala

## Diskussion

Die vorliegende Studie hatte zum Ziel, externe Zuweisungen zur EKT an unser Haus hinsichtlich (1) der Zuweiser, (2) der Patienten, (3) der vorangegangenen Pharmakotherapie und (4) des Therapieansprechens im Falle einer EKT-Behandlung zu untersuchen.

Während des hier analysierten einjährigen Zeitraums erreichten unsere Klinik 52 externe Anmeldungen zur EKT aus einem weitläufigen Gebiet um Göttingen herum (Median: 120 km). Die große Anzahl und der Zuweisungsradius weisen auf einen regional ungedeckten Bedarf an dieser evidenzbasierten Therapie hin. Selbst wenn ein aufnehmendes Zentrum verfügbar ist, bestehen auch dort limitierte Ressourcen: An der Universitätsklinik Göttingen entspräche die absolute Zahl der externen Anmeldungen einer neu beginnenden EKT-Serie pro Woche und somit nur für die Akutbehandlung jährlich mehr als 500 einzelnen Behandlungen zusätzlich. Darüber hinaus ist in vielen Fällen nach erfolgreicher Akutbehandlung eine Erhaltungs-EKT indiziert. Bei Patienten, die von extern zugewiesen werden, ist dies häufig mit hohem Aufwand und dem Zurücklegen weiter Strecken verbunden. In der hier vorliegenden Untersuchung erschienen einige Patienten trotz bestehender Aufnahmemöglichkeit aus unklaren Gründen nicht zur Aufnahme. Neben der teils weiten Entfernung zwischen Wohnort der Patienten und unserer Klinik könnte dies auch durch eine nachlassende Therapiemotivation durch zum Teil mehrmonatige Wartezeiten erklärbar sein. Trotz dieser Problematiken bleibt insgesamt klar positiv hervorzuheben, dass viele Behandler Patienten mit unter anderweitiger Therapie nicht ausreichend ansprechender Symptomatik an externe Kliniken verweisen.

Derzeit wird die Empfehlung zur EKT nach externer Zuweisung in unserem Haus durch auf dem Gebiet der EKT erfahrene Fachärzte für Psychiatrie und Psychotherapie ausgesprochen. Hier wird entschieden, ob eine EKT in unserem Haus geplant werden soll. In vielen Fällen erfolgt neben der Sichtung des Anmeldeformulars und ggf. weiterer Vorbefunde eine persönliche Rücksprache mit den ärztlich behandelnden Kollegen. Bei hinsichtlich der Indikation weiter unklaren Fällen wird eine stationäre Aufnahme im Vorfeld zur Mitbeurteilung des klinischen Zustands und Klärung des weiteren Prozederes vorangestellt. Dies könnte zukünftig sinnvoll durch einen regelhaft vorangestellten telemedizinischen Termin für die angemeldeten Patienten ergänzt werden.

Um flächendeckend EKT anbieten zu können, müsste dieses Therapieangebot dennoch zusätzlich an mehr psychiatrischen Kliniken etabliert werden.

Die Verteilung der Geschlechter (61,5 % weiblich) und das mittlere Patientenalter (M = 53,0) unserer Stichprobe entsprechen den Charakteristika anderer untersuchter EKT-Stichproben. Ungewöhnlich hoch war jedoch der Anteil von EKT-Anfragen für Patienten mit Störungen aus dem Schizophreniespektrum (36,5 %). Dementgegen wurden in Deutschland 2016 nur rund 18 % der EKT-Behandlungen aufgrund psychotischer Störungen durchgeführt (ca. 80 % aufgrund von Depressionen; [4]). Es ist anzunehmen, dass externe Verlegungen zur EKT vorwiegend bei den am schwersten betroffenen Patienten durchgeführt werden, was den relativ hohen Anteil an Patienten mit Schizophreniespektrumstörungen teilweise erklären könnte. Problematisch ist hierbei, dass andere Patienten mit weniger schwerwiegender Symptomatik, aber dennoch bestehender Indikation im Rahmen der externen Zuweisung potenziell un- oder unterversorgt bleiben.

Bezogen auf die Untersuchung der medikamentösen Vorgeschichte war zu erkennen, dass die meisten Patienten vor Anmeldung zur EKT mindestens eine leitliniengerechte Vorbehandlung erhielten. Dabei wurden erwartungsgemäß primär sogenannte „Soll“-Empfehlungen (Empfehlungsgrad A) sowie „Sollte“-Empfehlungen (Empfehlungsgrad B) umgesetzt. So konnte bei der Mehrzahl der unipolar depressiven Patienten ein Augmentationsversuch nachgewiesen werden (B), bei der Mehrzahl der Patienten mit Schizophreniespektrumstörungen ein Therapieversuch mit Clozapin (A). Häufig war aber nicht nachvollziehbar, ob Medikamente in adäquater Dosierung und ausreichender Länge gegeben wurden. Eine mögliche Erklärung hierfür sind fehlende Informationen zur Vorgeschichte bedingt durch eine zumeist langjährige Krankheitsgeschichte sowie wechselnde ambulante und stationäre Vorbehandlungen. Die zahlreichen vorangegangenen Therapieversuche (im Mittel mehr als 5 Antidepressiva/mehr als 5 Antipsychotika) zeigen, dass die EKT häufig erst zu einem späten Zeitpunkt im Therapieverlauf erwogen wurde, obwohl klinische sowie gesundheitsökonomische Daten für einen frühzeitigeren Einsatz sprechen [20, 21]. In diesem Kontext soll erwähnt werden, dass vor Indikationsstellung zur EKT nicht sämtliche sonstigen Therapieempfehlungen erfüllt sein müssen und dass Gründe wie zum Beispiel Unverträglichkeit der Pharmakotherapie oder eine entsprechende Patientenpräferenz ein Abweichen von Leitlinienempfehlungen rechtfertigen.

Die zahlreichen unwirksamen Vorbehandlungen sowie der überwiegende Anteil von stationär behandelten Patienten zum Zuweisungszeitpunkt spiegeln die hohe Krankheitsschwere in der hier vorliegenden Stichprobe wider. Das gute Therapieansprechen ist für diese Patienten, die zum Teil erst nach langen Wartezeiten eine EKT erhielten, besonders hervorzuheben. Für affektive Erkrankungen ist belegt, dass eine hohe Anzahl vorangegangener erfolgloser Therapieversuche und eine längere Krankheitsdauer negative Prädiktoren für ein positives Therapieansprechen darstellen [22, 23]. Dennoch fand sich in unserer Untersuchung in allen Diagnosegruppen bei 72,2 % der Patienten eine starke bis sehr starke Zustandsverbesserung. Dies entspricht in etwa den Ergebnissen aus kontrollierten Studien (z. B. [24–26]).

### Limitationen

Die häufig unvollständig aufgeführten Patientenanamnesen zeigen die Schwierigkeit, gerade bei langen Krankenvorgeschichten alle Vorbehandlungen zusammenzutragen. Entsprechend waren die hier berichteten Daten in vielen Fällen lückenhaft, was eine deskriptive Auswertung erschwert und eine inferenzstatistische Auswertung nicht sinnvoll erscheinen lässt. Häufig fanden Vorbehandlungen in verschiedenen Praxen und Kliniken statt. Eine Möglichkeit zur Optimierung stellt hier unter Umständen die Etablierung einer digitalen und behandlerübergreifenden Krankenakte dar. Für uns stellt sich die Frage, ob eine neue Struktur des Anmeldeformulars zum Beispiel mittels Erfassung von Vorbehandlungen durch Auswahlangaben den Prozess für die Zuweiser erleichtern könnte.

Weiterhin handelt es sich hier um eine rein retrospektive Analyse. Aufgrund der kleinen Fallzahl war eine Aufschlüsselung der Fälle zum Beispiel nach verschiedenen Diagnosen nicht sinnvoll. Die Zuweisung durch verschiedene Behandler bedingte zudem eine heterogene Handhabe in Bezug auf den Zeitpunkt der Zuweisung und die Darstellung der Vorgeschichte der Patienten – insgesamt müssen die hier gefundenen Ergebnisse somit zurückhaltend interpretiert werden. Ferner sollte beachtet werden, dass die Extraktion von CGI-I-Werten aus Arztbriefen ein weniger exaktes Maß der therapeutischen Verbesserung ist als etwa syndromspezifische Schweregradskalen.

## Fazit für die Praxis

Regional besteht ein hoher ungedeckter Bedarf an EKT zur Behandlung schwerer und therapieresistenter Störungen, vor allem aus dem affektiven sowie Schizophreniespektrum. Dieser Bedarf kann nur teilweise durch Verlegungen gedeckt werden – dies verdeutlicht den limitierten Zugang vieler Patienten zu einer evidenzbasierten und hochwirksamen Therapie. Regional fehlende Angebote führen spätestens nach Entlassung aus der Akutbehandlung zu logistischen Folgeproblemen in der EKT-Erhaltungstherapie. Gerade vor dem Hintergrund limitierter Ressourcen ist vor Indikationsstellung zur EKT eine lege artis durchgeführte Pharmakotherapie essenziell. Positiv hervorzuheben ist, dass trotz zum Teil langer Wartezeiten der von extern zugewiesenen Patienten die Ansprechraten auf die EKT hoch sind und sich auf dem Niveau von kontrollierten Studien bewegen.
